# Localised labyrinthine patterns in ecosystems

**DOI:** 10.1038/s41598-021-97472-4

**Published:** 2021-09-15

**Authors:** M. G. Clerc, S. Echeverría-Alar, M. Tlidi

**Affiliations:** 1 Departamento de Física and Millennium Institute for Research in Optics, Facultad de Ciencias Físicas y Matemáticas, Universidad de Chile, Casilla 487-3, Santiago, Chile; 2 Département de Physique, Faculté des Sciences, Université Libre de Bruxelles (U.L.B.), CP 231, Campus Plaine, 1050 Brussels, Belgium

**Keywords:** Ecology, Physics, Applied physics, Biophysics

## Abstract

Self-organisation is a ubiquitous phenomenon in ecosystems. These systems can experience transitions from a uniform cover towards the formation of vegetation patterns as a result of symmetry-breaking instability. They can be either periodic or localised in space. Localised vegetation patterns consist of more or less circular spots or patches that can be either isolated or randomly distributed in space. We report on a striking patterning phenomenon consisting of localised vegetation labyrinths. This intriguing pattern is visible in satellite photographs taken in many territories of Africa and Australia. They consist of labyrinths which is spatially irregular pattern surrounded by either a homogeneous cover or a bare soil. The phenomenon is not specific to particular plants or soils. They are observed on strictly homogenous environmental conditions on flat landscapes, but they are also visible on hills. The spatial size of localized labyrinth ranges typically from a few hundred meters to ten kilometres. A simple modelling approach based on the interplay between short-range and long-range interactions governing plant communities or on the water dynamics explains the observations reported here.

## Introduction

The appearance of order and structures that involve nonequilibrium exchanges of energy and/or matter have been widely observed in many natural systems including fluid mechanics, optics, biology, ecology, and medicine^1–6^. Vegetation populations and vegetation patterns belong to this field of research. Being often undetectable at the soil level, large-scale vegetation patterns have been first observed thanks to the usability of aerial photographs in the early fifties^7^. They appear as extended bands of vegetation alternating periodically with vegetated areas and unvegetated bands. These large-scale botanical organisations have been reported in many semi-arid and arid ecosystems of Africa, Australia, America, and Asia. It is now widely admitted that the origin of these large scale botanical organisations is attributed to a nonequilibrium symmetry-breaking instability leading to the establishment of stable periodic spatial patterns. Extended and periodic vegetation pattern arising in semi-arid and arid ecosystems has been the subject of numerous studies and is by now fairly well-understood issue (see recent overviews^8–10^ and references therein).

Vegetation patterns are not always periodic and extended in space. They can be spatially localised and aperiodic consisting of isolated or randomly distributed patches on bare soil^11–13^ or gaps embedded in a uniform vegetation cover^14,15^. They are generated in a regime where the homogeneous cover coexists with periodic vegetation patterns. The interaction between well-separated patches is always repulsive^16,17^ while for gaps the interaction alternates between attractive and repulsive depending on the distance separating gaps^14,17^. The localised patches has a more or less circular shape. However, for a moderate aridity condition, the circular shape can exhibit deformation followed by splitting of a single into two new patches. Newer patches will in their turn exhibit deformation and self-replication^18–20^ until the system reaches a periodic distribution of patches that occupies the whole space available in landscapes^19,20^. This process leading to spotted periodic patterns can be seen as warnings of ecosystem degradation and may lead to outcome of vegetation recovery. Besides patches self-replication, circular spots can exhibit deformation leading to the formation of arcs and spirals like in isotropic and uniform environmental conditions^21^. The vegetation spirals are not waves since they do not rotate^21^.

**Figure 1:**
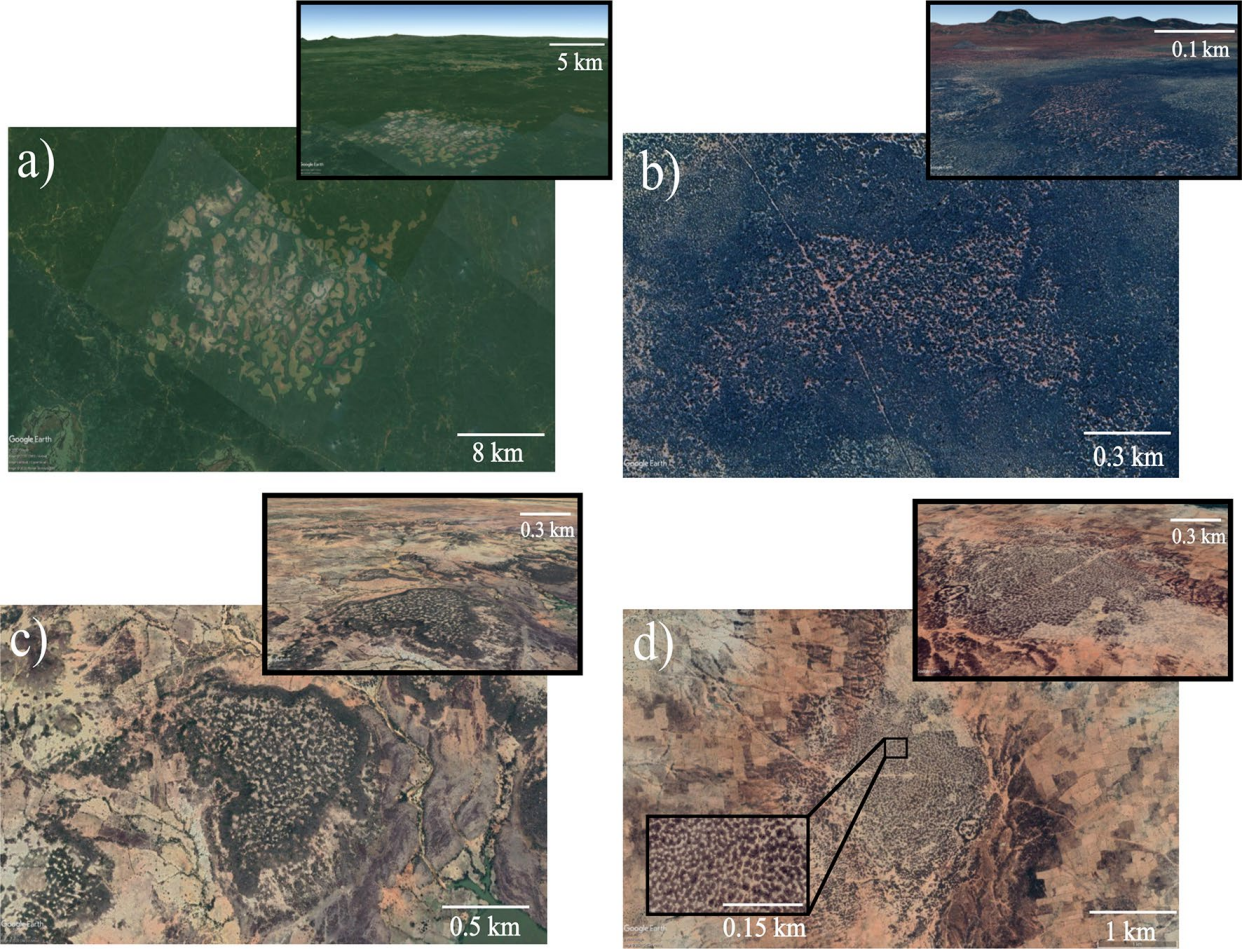
Localised labyrinth vegetation patterns. Top views of (**a**) Central Cameroon (3$^\circ$ 59′ 22.05″ N 12$^\circ$ 17′ 20.99″ E), (**b**) Western Australia (29$^\circ$ 33′ 36.16″ S 117$^\circ$ 15′ 32.60″ E), (**c**) and (**d**) Southwest Niger (12$^\circ$ 34′ 45.10″ N 2$^\circ$ 41′ 28.71″ E and 12$^\circ$ 22′ 6.72″ N 3$^\circ$ 28′ 39.35″ E, respectively). The inset (d) show a zoom of the characteristic labyrinth pattern. All the images were retrieved from Google Earth software (https://earth.google.com/web/) with a resolution of $1920\times 1080$ pixels (total areas of (**a**) 196.5 $\hbox {km}^{2}$, (**b**) 7.4 $\hbox {km}^{2}$, (**c**) 12.3 $\hbox {km}^{2}$, and (**d**) 24.6 $\hbox {km}^{2}$). The satellite images were taken on 17 of February, 2021; 22 of September, 2018; 15 of November, 2016; and 12 of February, 2020, respectively. The upper-right insets show the localised patterns to emphasize the topography of the landscape.

In this work, we unveil a new type of vegetation pattern consisting of a localised labyrinth embedded either in a homogeneous cover or surrounded by bare soil. This phenomenon is observed in Africa and Australia by remote sensing imagery. An example of such a botanical self-organisation phenomenon is shown in Fig. 1. They consist of either an irregular distribution of vegetation surrounded by a uniform cover (see Fig. 1a, b), or by a bare state (Fig. 1c, d). We show that localised labyrinths embedded in a uniform cover can be stable even if the environment is isotropic and their formation does not depend on the topography. However, when a localised labyrinth is surrounded by a bare state, they can expand or shrink. We analyse this phenomenon by using three well-known self-organisation vegetation models, which support localised labyrinths. We show that localised labyrinths are permanent structures, and they can be observed worldwide involving a range of species and spatial scales. We interpret this phenomenon as a spatial compromise between the extended labyrinth that occupies the whole space available and stable homogeneous states. More precisely, the mechanism leading to their formation is attributed to the pinning-depinning transition that takes place in a parameter space where models exhibit bistability between extended disordered pattern and homogeneous cover.

## Field observations of localised labyrinths

**Figure 2:**
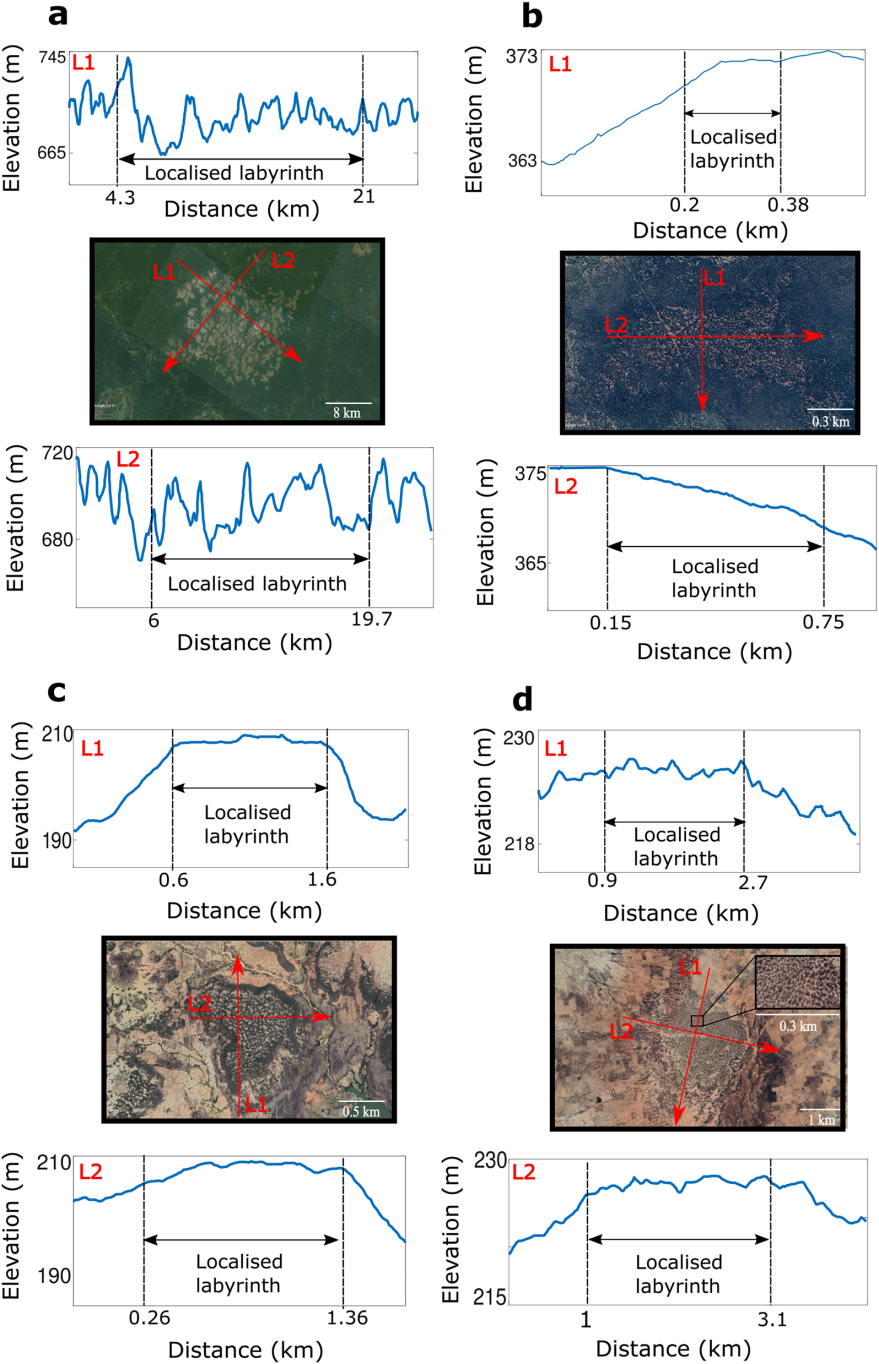
Elevation profiles of the localised labyrinths observed in nature (Fig. 1). They were obtained using Google Earth software (https://earth.google.com/web/). In each zone two elevation profiles are shown for two arbirtary cross-sections (L1 and L2). (**a**) The localised labyrinth in central Cameroon has large fluctuations in height ranging from 665 to 745 m. The homogenous cover that surrounds the localised labyrinth also has fluctuations in height of the same order. The size of the major axis of the localised pattern is 16.7 km. (**b**) In Western Australia the localised labyrinth is in a gentle slope ($0.8\%$), the size of its major axis is 0.6 km. (**c**) and (**d**) shows the elevation profiles of the localised labyrinths in Southwest Niger. Both patterns are in small hills of about 10 m, surrounded by a bare state. These localised labyrinths emerge in plain terrains. The sizes of the major axes are 1.0 km and 2.0 km, respectively. See the "Methods" section for details on the accuracy of the elevation data.

Localised labyrinths observed in nature are large-scale self-organisation patterns. They are satellite images from Africa and Australia obtained by the use of Google Earth software. The landscape of Central Cameroon (zone of forest-savanna mosaic^22^), shown in Fig. 1a, displays contrasted phases of bare and densely vegetated areas with well-defined scale and symmetry surrounded by more or less uniform woodland. The climate in the zone where we observe the localised labyrinth is humid, with annual averaged precipitation of 1800 mm^23^. The annual averaged of potential evapotranspiration is between 1500 and 1600 mm^24^. The localised labyrinth we observe in Western Australia (see Fig. 1b) consists of localised woodland embedded in the shrubland of Mulga Bush (Acacia Aneura)^25^. In this zone the climate is arid, where the mean annual precipitation is 250 mm^26^ and the mean annual potential evapotranspiration is between 1200 and 1300 mm^27^. Besides, the localised labyrinth can be surrounded by bare zones as shown in landscapes of Southwest Niger in a brush-grass Savanna zone^28^ (Fig. 1c, d). In this region the climate is semi-arid, the mean annual rainfall is 605 mm, in between June and September^29^, and the annual mean potential evapotranspiration near this zone is 1900 mm^30^. All the climate data is summarized in Table 1 in Methods section. Sparsely populated or bare areas alternate with dense vegetation irregular bands or patches made of microchloa Indica. The field observations suggest that localised labyrinthine structures are formed both in a flat landscape and with topographic variation (see Fig. 2). By their spatial regularity, by their spatial scales ranging from a few hundred meters to ten kilometres, as well as by the composition of their vegetation (tree, shrubs, herbs, and grasses), localised labyrinthine patterns are permanent structures, and they can be observed even in non-arid climates. They have neither been observed nor reported. Understanding their formation and maintenance is an important ecological issue.

**Table 1:** Mean annual precipitation, potential evapotranspiration, and aridity index of the regions where localised labyrinthine patterns are observed.

	Precipitation (mm)	Potential evapotranspiration (mm)	Aridity index	Classification
Central Cameroon	1800	1500–1600	1.1–1.2	Humid
Western Australia	250	1200–1300	0.1–0.2	Arid
Southwest Niger	605	1900	0.3	Semi-arid

For more details on the meteorological data see the references given in the text.

The mechanism underlying the emergence of the localised labyrinth can be captured by using self-organisation mathematical models that can explain vegetation pattern formation within a unified conceptual framework. In this respect, two approaches will be used. The first is based on the relationship between the plants’ aerial-subterranean structures, the facilitative and competitive feedbacks which act at the community level, and the plants’ spatial propagation by seed dispersion^31,32^. The second approach incorporates explicitly water transport by below ground diffusion and/or above ground run-off^33–35^. These models are in reasonable agreement with the field observations^36–38^.

## Mathematical modelling of ecosystems

The absence of the first principles for biological systems in general, and in particular for vegetation populations where phenomena are interconnected makes their mathematical modelling complex. The theory of vegetation pattern formation rests on the self-organisation hypothesis and symmetry-breaking instability that provoke the fragmentation of the uniform cover. The symmetry-breaking instability takes place even if the environment is isotropic^31,33,35^. This instability may be an advection-induced transition that requires the pre-existence of the environment anisotropy due to the topography of the landscape^34,39,40^. Generally speaking, this transition requires at least two feedback mechanisms having a short-range activation and a long-range inhibition. In this respect, we consider three different vegetation models that are experimentally relevant systems: (i) the generic interaction redistribution model describing vegetation pattern formation which incorporates explicitly the facilitation, competition and seed dispersion nonlocal interactions (ii) the local nonvariational partial differential model described by a nonvariational Swift–Hohenberg type of model equation, and (iii) the reaction–diffusion system that incorporate explicetely water transport.

### The interaction-redistribution approach

#### The integrodifferential model

This approach consists of considering a well-known logistic equation with nonlocal plant-to-plant interactions. Three types of interactions are considered: the facilitative $M_{f}(\mathbf {r},t)$, the competitive $M_{c}(\mathbf {r},t)$, and the seed dispersion $M_{d}(\mathbf {r},t)$ nonlocal interactions. To simplify further the mathematical modelling, we consider that the seed dispersion obeys a diffusive process $M_{d}(\mathbf {r},t)\approx \nabla ^{2}b(\mathbf {r},t)$, with *D* the diffusion coefficient, *b* the biomass density, and $\nabla ^{2}=\partial ^2/\partial x^2+\partial ^2/\partial y^2$ is the Laplace operator acting in the (*x*,*y*) plane. The interaction-redistribution reads

1 $$\begin{aligned} M_{i}=exp\left\{ \frac{\xi _{i}}{N_{i}}\int b(\mathbf {r}+\mathbf {r}',t)\phi _i(r,t)d\mathbf {r}'\right\} , { \text{ with } } \phi _i(r,t)= exp(-r/L_{i}) \end{aligned}$$

where $i=f,c$. $\xi _i$ represents the strength of the interaction, $N_i$ is a normalisation constant. We assume that their Kernels $\phi _i(r,t)$ are exponential functions with $L_i$ the range of their interactions. The facilitative interaction $M_{f}(\mathbf {r},t)$ favouring vegetation development. They involve the accumulation of nutrients in the neighbourhood of plants, the reciprocal sheltering of neighbouring plants against climatic harshness which improves the water budget in the soil. The range of the facilitative interaction $L_f$ operates on the crown size. The competitive interaction operates over a length $L_c$ and involves the below-ground structures, i.e., the rhizosphere. In nutrient-poor or/and in water-limited territories, lateral spreading may extend beyond the radius of the crown. This extension of roots relative to their crown size is necessary for the survival and the development of the plant in order to extract enough nutrients and/or water from the soil. When incorporating these nonlocal interactions in the paradigmatic logistic equation, the spatiotemporal evolution of the normalised biomass density $b(\mathbf {r}, t)$ in isotropic environmental conditions reads^14^

2 $$\begin{aligned} \partial _{t} b(\mathbf {r},t)=b(\mathbf {r},t)[1-b(\mathbf {r},t)]M_{f}(\mathbf {r},t)- \mu b(\mathbf {r},t)M_{c}(\mathbf {r},t)+D\nabla ^{2}b(\mathbf {r},t). \end{aligned}$$

The normalisation is performed with respect to the total amount of biomass supported by the system. The first two terms in the logistic equation with nonlocal interaction Eq. (2) describe the biomass gains and losses, respectively. The third term models seed dispersion. The aridity parameter $\mu$ accounts for the biomass loss and gain ratio, which depends on water availability and nutrients soil distribution, topography, etc. The homogeneous cover solutions of Eq. (2) are: $b_{o}=0$ which corresponds to the state totally devoid of vegetation, and the homogeneous cover solutions satisfy the equation

3 $$\begin{aligned} \mu =(1-b)\exp (\Delta b), \end{aligned}$$

with $\Delta =\xi _{f}-\xi _{c}$ measures the community cooperativity if $\Delta >0$ or anti-cooperativity when $\Delta <0$. The bare state $b_{o}=0$ is unstable (stable) $\mu <1$ $(\mu >1$). The homogeneous cover state with higher biomass density is stable and the other is unstable. These solutions are connected by a saddle-node or a tipping point whose coordinates are given by $\left\{ b_{sn}=(\Delta -1)/\Delta ,\mu _{sn}=e^{\Delta -1}/\Delta \right\}$. The linear stability analysis of vegetated cover ($b_{s}$) with respect to small fluctuations of the from $b(\mathbf {r},t)=b_{s}+ \delta b\ exp\{\sigma t+i\mathbf {k}\cdot \mathbf {r}\}$ with $\delta b$ small, yields the dispersion relation

4 $$\begin{aligned} \sigma (k)=\left( b_{s}(1-b_{s})\xi _{f}-b_{s}-\frac{b_{s}(1-b_{s})\xi _{c}}{(1+L_{c}^{2}k^{2})^{3/2}}\right) e^{\xi _{f}b_{s}}-Dk^{2}. \end{aligned}$$

Given the spatial isotropy, the growth rate $\sigma (k)$ is a real quantity. This eigenvalue may become positive for a finite band of unstable modes which triggered the spontaneous amplification of spatial fluctuations towards the formation of periodic structures with a well-defined wavelength. At the symmetry-breaking instability the value of the critical wavenumber $k_c$ marking the appearance of a band of unstable modes, and hence the symmetry-breaking instability, can be evaluated by two conditions: $\sigma (k_c)=0$ and $\partial \sigma /\partial k|_{k_{c}}=0$. These conditions yield the most unstable mode

5 $$\begin{aligned} k_{c}^{2}=\frac{1}{L_{c}^{2}}\left[ \left( \frac{3b_{s}e^{\xi _{f}b_{s}}(1-b_{s})\xi _{c}L_{c}^{2}}{2D}\right) ^{2/5}-1\right] . \end{aligned}$$

This critical wavenumber determines the wavelength of the periodic vegetation pattern $2\pi /k_c$ that emerges from the symmetry-breaking instability. Replacing $k_c$ in the condition $\sigma (k_{c})=0$, we can then calculate the critical biomass density $b_{c}$. The corresponding critical aridity parameter $\mu _{c}$ is provided explicitly by the homogeneous steady states Eq. (3).

#### Local model: a nonvariational Swift–Hohenberg model

The integrodifferential equation (2) can be reduced by means of a multiple-scale analysis to a simple partial differential equation, in the form of nonvariational Swift–Hohenberg equation. This reduction has been performed in the neighbourhood of the critical point associated with the nascent bistability^14,32^. The coordinates of the critical point are: the biomass density $b_c = 0$, the cooperativity parameter $\Delta _c=1$, and the aridity parameter $\mu _c=1$. These coordinates are obtained from Eq. (3) by satisfying the double condition $\partial \mu /\partial b_s=0$ and $\partial ^2\mu /\partial b_s^2=0$. To apply a multiple-scale analysis it is necessary to define a small parameter that measures the distance from criticality and expand *b*, $\mu$, and $\Delta$ in the Taylor series around their critical values. The symmetry-breaking instability should be close to that critical point. To fulfil this condition, we must consider a small diffusion coefficient in order to include the symmetry-breaking instability in the description of the dynamics of the biomass density. This reduction is valid in the double limit of nascent bistability and close to the symmetry-breaking instability. In this double limit, the time-space evolution of biomass density obeys a non-variational Swift–Hohenberg model^14^

6 $$\begin{aligned} \partial _{t}u(\mathbf {r},t)=-u(\mathbf {r},t)(\eta -\kappa u(\mathbf {r},t)+u(\mathbf {r},t)^{2})+\left[ \nu -\gamma u(\mathbf {r},t)\right] \nabla ^{2}u(\mathbf {r},t) -\alpha u(\mathbf {r},t)\nabla ^{4}u(\mathbf {r},t), \vspace{0.3cm} \end{aligned}$$

where $\eta$ and $\kappa$ are, respectively, the deviations of the aridity and cooperativity parameters from their values at the critical point. The linear and nonlinear diffusion coefficients $\nu$, $\gamma$, and $\alpha$ depend on the shape of kernels^17^. In addition to the bare state $u=0$, the homogeneous covers obey

7 $$\begin{aligned} u_{\pm }=\frac{\kappa \pm \sqrt{\kappa ^{2}-4\eta }}{2}, \end{aligned}$$

where the two homogeneous solutions $u_{\pm }$ are connected through the saddle-node bifurcation $\left\{ u_{sn}=\kappa /2,\eta _{sn}=\kappa ^{2}/4\right\}$, with $\kappa >0$. The solution $u_{-}$ is always unstable even in the presence of small spatial fluctuations. The linear stability analysis of vegetated cover ($u_{+}$) with respect to small spatial fluctuations, yields the dispersion relation

8 $$\begin{aligned} \sigma (k)=u_{+}(\kappa -2u_{+})-(\nu -\gamma u_{+})k^{2}-\alpha u_{+}k^{4}. \end{aligned}$$

Imposing $\partial \sigma /\partial k|_{k_{c}}=0$ and $\sigma (k_{c})=0$, the critical mode can be determined

9 $$\begin{aligned} k_{c}=\sqrt{\frac{\gamma -\nu /u_{c}}{2\alpha }}, \end{aligned}$$

where $u_{c}$ satisfies $4\alpha u_{c}^2(2u_{c}-\kappa )=(2\gamma u_{c}-\nu )^2$. The corresponding aridity parameter $\eta _{c}$ can be calculated from Eq. (7).

### The reaction–diffusion approach

The second approach explicitly adds the water transport by below ground diffusion. The coupling between the water dynamics and the plant biomass involves positive feedbacks that tend to enhance water availability. Negative feedbacks allow for an increase in water consumption caused by vegetation growth, which inhibits further biomass growth.

The modelling considers the coupled evolution of biomass density $b(\mathbf {r},t)$ and groundwater density $w(\mathbf {r},t)$. In its dimensionless form, this model reads^33^

10 $$\begin{aligned} \frac{\partial b}{\partial t}= & {} \frac{\gamma w}{1+\omega w}b-b^{2}-\theta b+\nabla ^{2}b, \end{aligned}$$

11 $$\begin{aligned} \frac{\partial w}{\partial t}= & {} p-(1-\rho b)w-w^{2}b+\delta \nabla ^{2}(w-\beta b). \end{aligned}$$

The first term in the first equation describes plant growth at a constant rate ($\gamma /\omega$) that grows linearly with *w* for dry soil. The quadratic nonlinearity $-b^{2}$ accounts for saturation imposed by poor nutrients soil. The term proportional to $\theta$ accounts for mortality, grazing or herbivores. The mechanisms of dispersion are modelled by a simple diffusion process. The groundwater evolves due to a precipitation input *p*. The term $(1-\rho b)w$ in the second equation accounts for the evaporation and drainage, that decreases with the presence of vegetation. The term $w^{2}b$ models the water uptake by the plants due to the transpiration process. The groundwater movement follows the Darcy’s law in unsaturated conditions; that is, the water flux is proportional to the gradient of the water matric potential^41^. The matric potential is equal to *w*, under the assumption that the hydraulic diffusivity is constant^41^. To model the suction of water by the roots, a correction to the matric potential is included; $-\beta b$, where $\beta$ is the strength of the suction.

## Results

### Localised labyrinthine vegetation pattern

**Figure 3:**
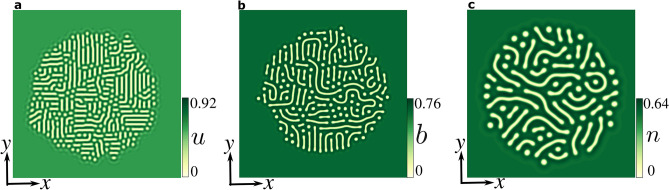
Numerical observations of localised labyrinths. The model-independent structure is observed in (**a**) a non-variational Swift–Hohenberg model, (**b**) integrodifferential non-local model, and (**c**) reaction–diffusion model. In the three cases the labyrinth is supported by a uniform vegetated state. The parameters used in each model are listed in the "Methods" section. From numerical simulations, the figure was created using Inkscape 1.0 (https://inkscape.org/release/inkscape-1.0/).

In our analysis, we focus on the simplest vegetation model that has been derived from the interaction-redistribution approach, namely the non-variational Swift–Hohenberg Eq. (6) described above. This model is appropriate to describe the space-time dynamics of the biomass under resource-limited landscapes such as nutrient limitation or water deprivation. In this case, the average biomass density is low comparing the carrying capacity closed-packing density of unstressed vegetation. The simulated stationary localised vegetation labyrinth is shown in Fig. 3a. Moreover, to confirm the field observation and to show that this phenomenon is model-independent, we conducted numerical simulations of the other two models, the integrodifferential (Eq. (2)) based on the facilitative, competitive, and seed dispersion interactions; and the reaction–diffusion type that explicitly incorporates water transport (Eq. (11)). The results are shown in Fig. 3b and 3c. The parameters used to simulate the different localised labyrinths are listed in Tables 2, 3, and 4 in the "Methods" section. The localised labyrinth consists of one spatially disordered state surrounded by a qualitatively different state. Note that the localised labyrinthine patterns shown in Fig. 3 do not have a round shape. The fact that this shape is not round is attributed to the presence of defects in the disordered pattern since they modify the interface energy. Investigations of fronts propagation between labyrinths and homogeneous states mediated by defects are missing in the literature. The interface separating these two states is stationary leading to a fixed size of a localised labyrinth. It neither grows and invades the uniform cover nor shrinks. The stabilization of localised labyrinth is attributed to the interface pinning phenomenon^42,43^. This phenomenon is characterized by an interface that connects a homogeneous state and a periodic one, which is motionless on a finite region of parameters, pinning range. This pinning effect occurs due to the competition between a global energy symmetry breaking between states that favors the interface propagate in one direction and the spatial modulations that block the interface by introducing potential barriers^42^.

**Figure 4:**
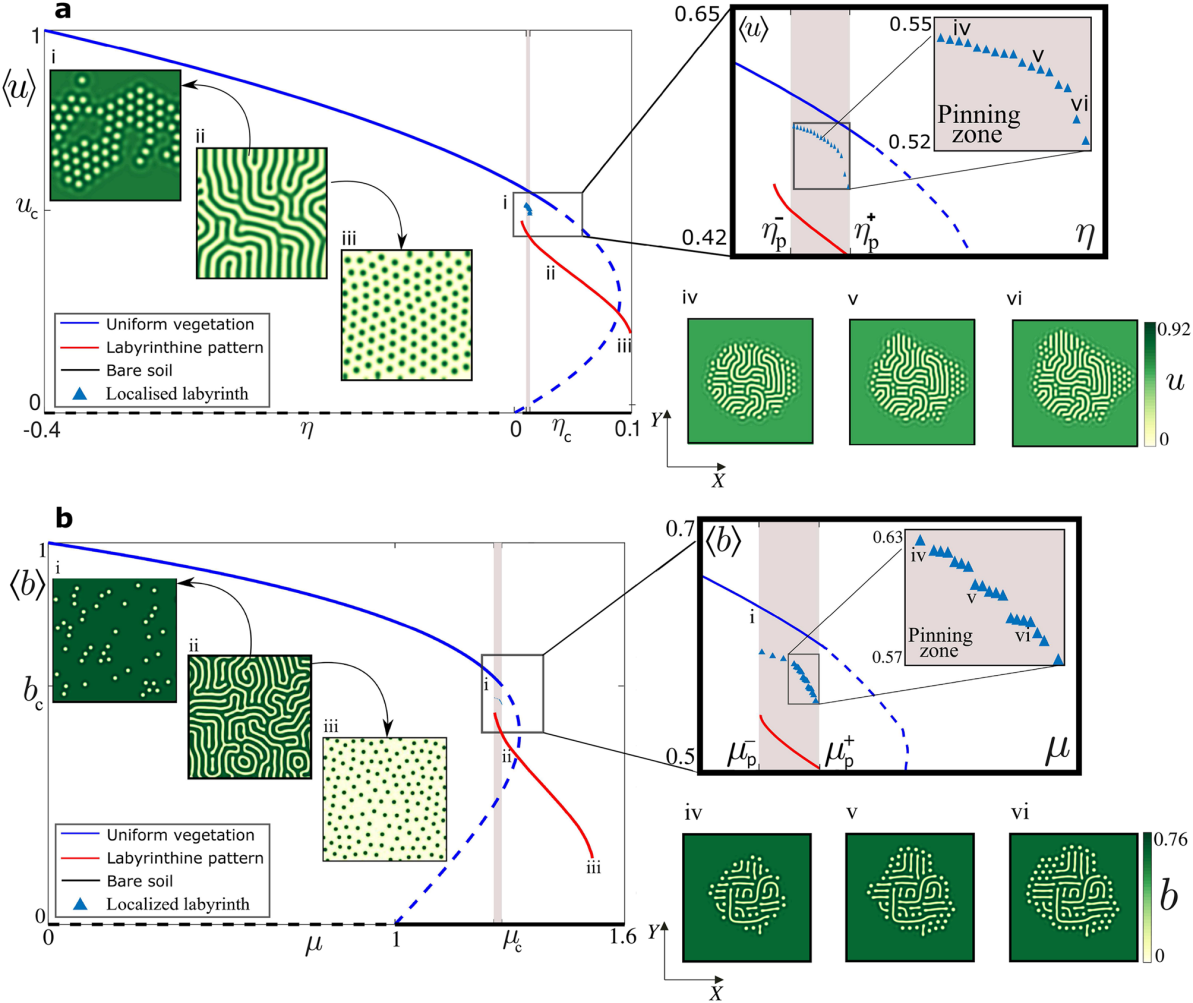
Bifurcation diagram of vegetation models. (**a**) the non-variational Swift–Hohenberg model, and (**b**) the integrodifferential model. Gaps (i), labyrinths (ii), and spots (iii) is the standard sequence of patterns in vegetation models. $\left\langle u\right\rangle$ and $\left\langle b\right\rangle$ stands for the average biomass in each model. The solid curves indicate where the bare soil or uniform vegetation cover are stable, whereas the segmented curves indicate where these states are unstable. In (**a**), the critical point $(\eta _{c}=0.038,u_{c}=0.53)$ stands for the instability threshold where the uniform vegetated cover loses stability to a modulated state. In a narrow region, between $\eta _{p}^{-}=0.010$ and $\eta _{p}^{+}=0.013$, where there is a multistability of states (labyrinth, uniform vegetation, bare soil) the emergence of localized labyrinths is possible. In (**b**), $(\mu _{c}=1.309,b_{c}=0.62)$ and $\mu _{p}^{-}=1.2950$, $\mu _{p}^{+}=1.3044$. The insets with the pinning zones enlarged show the existence of a family of localized labyrinths (triangles) with different average biomasses. The insets (iv), (v), and (vi) show different localized labyrinthine patterns [(**a**) and (**b**)]. The other parameters are provided in the "Methods" section. From numerical simulations, the figure was created using Inkscape 1.0 (https://inkscape.org/release/inkscape-1.0/).

To determine the stability domain of the localised labyrinth, we establish the bifurcation diagram shown in Fig. 4a, where we plot the biomass density as a function of the aridity parameter $\eta$. The aridity refers not only to water scarcity but can be also attributed to the nutrient-poor soil. When the aridity is low obviously the uniform vegetated state is stable (blue line) and the bare state (broken line) is unstable. When the aridity parameter is further increased, the homogeneous cover becomes unstable with respect to small fluctuations. Above this symmetry-breaking instability, several branches of solutions emerge sub-critically for $\eta <\eta _c$. Example of vegetation patterns that appears follows the well-known sequence made sparse vegetation spots that can be either periodic or localised in space (see i, Fig. 4a), banded vegetation (see ii, Fig. 4a) or a periodic distribution of localised patches setting on the bare state (see iii, Fig. 4a).

An extended labyrinthine pattern can be generated subcritically as indicated by the red line in the bifurcation diagram (see Fig. 4a). The situation which interests us requires that this extended labyrinth exhibits a coexistence with the uniform vegetated state. The coexistence between these two qualitatively different states is the prerequisite condition for the formation of a stable localised labyrinth. However, this condition is necessary but not sufficient, the interface separating these two states exhibits a pinning phenomenon^42^. Indeed, as shown in the inset of Fig. 4a, there exists a finite range of the aridity parameter often called the pinning zone $\eta _p^{-}<\eta <\eta _p^{+}$, where localised labyrinthine patterns are stable. Examples of localised labyrinth obtained by numerical simulations for fixed values of the control parameters are shown in Fig. 4a (iv, v, vi). The motionless interface is not necessarily circular, and contains bands perpendicular to it and circular patches. Similar bifurcation diagram is obtained from the integrodifferential model (see Fig. 4b).

**Figure 5:**
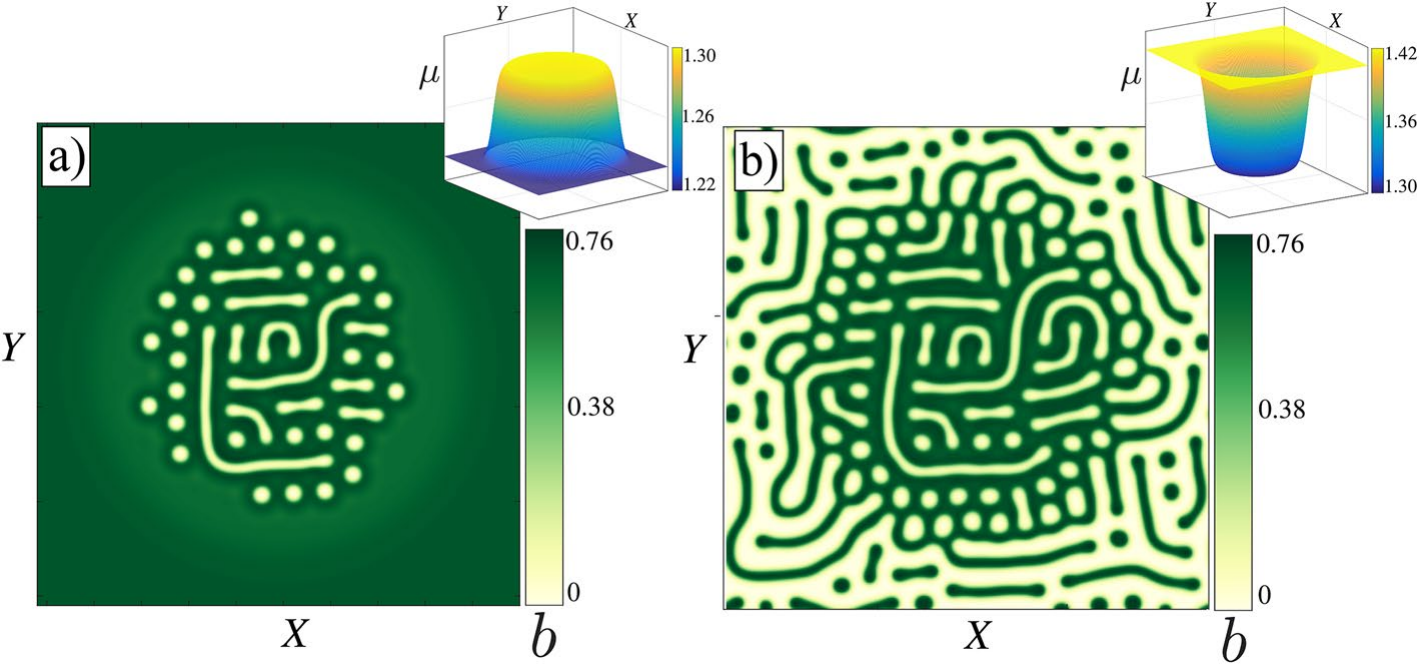
Localised labyrinthine patterns generated by inhomogenous aridity in the integrodifferential model. The spatially forced pattern can be supported by (**a**) the vegetated state (top hat-like shape $\mu$ parameter), and (**b**) the bare state (inverted top hat-like shape $\mu$ parameter). From numerical simulations, the figure was created using Inkscape 1.0 (https://inkscape.org/release/inkscape-1.0/).

Finally, we discuss the situation where the aridity is not homogenous due to the topography. For this purpose, we choose a top hat-like shape for the aridity parameter as shown in Fig. 5a. In this case, numerical simulations of the integrodifferential model Eq. (2) show a stable localised labyrinthine pattern (see Fig. 5a). Note that the localised labyrinthine structures surrounded by bare soil shown in Fig. 1c, d are unstable since the interface propagates. The interface can not be pinned in the absence of spatial oscillations around the bare state. Oscillations around this state are unphysical since the biomass density is a positive defined quantity. However, when the aridity parameter possesses an inverted top hat-like shape, it is possible to pin the interface (see Fig. 5b). In this case, the localised labyrinthine pattern is surrounded by a mosaic extended state, and the mechanism of stabilization is rather due to the inhomogeneity of the aridity parameter.

### Deppining mechanism

**Figure 6:**
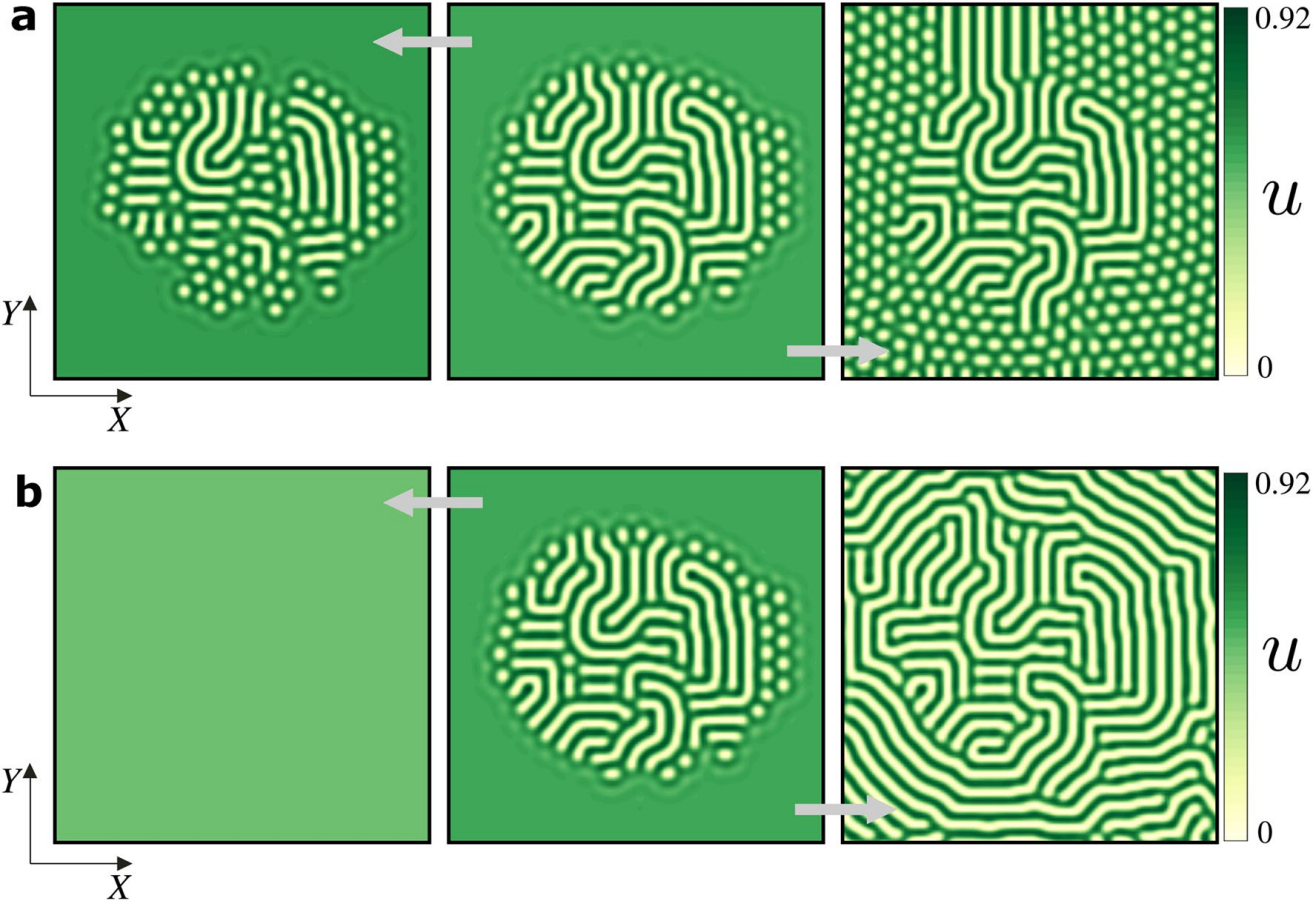
Deppining transitions of a localised labyrinth state ($\eta =0.0102$) in a non-variational Swift–Hohenberg model. This state is shown in the middle panel of (**a**) and (**b**). The localised pattern destabilize when crossing the pinning region boundaries when varying slowly (**a**) or abruptly (**b**) the aridity parameter. In the first case (**a**), when decreasing $\eta$ the localized labyrinth loses its internal structure due to shrinking of stripes (left panel, $\eta =0.007$), and when increasing $\eta$ some stripes begin to grow at the interface of the localized labyrinth and a hexagon pattern starts to invade the uniform cover (right panel, $\eta =0.016$) . In the second case (**b**), when decreasing $\eta$ all the stripes and patches of sparse vegetation disappear in favor of a uniform vegetated cover (left panel, $\eta =-0.03$), and when increasing $\eta$ the vegetated cover becomes unstable and stripes emerge. This process transform the localised labyrinth into an extended one (right panel, $\eta =0.05$). The other parameters are provided in the "Methods" section. From numerical simulations, the figure was created using Inkscape 1.0 (https://inkscape.org/release/inkscape-1.0/).

The spatial location of the localised labyrinth immersed in the bulk of the stable uniform vegetated state depends on the initial condition considered. When ecosystems operate out of the pinning zone, the interface separating the labyrinth and the homogeneous cover propagates due to the depinning transition (see Fig. 6a, b). In this case, depending on the aridity level, the interface propagates from one stable state to another The transition is different when moving the aridity parameter slowly or abruptly. In the second type of variation, when $\eta <\eta _p^{-}$, the homogeneous cover invades the system, while when $\eta >\eta _p^{+}$, the localised labyrinth survives but it is now embedded by a periodic distribution of gaps (see Fig. 6b).

## Conclusions

In this paper we have reported for the first time evidence of localised labyrinthine vegetation patterns observed on satellite images from Africa and Australia. We have shown that these localised structures are robustly consisting of either an irregular distribution of vegetation surrounded by a uniform cover or on the contrary surrounded by a bare state. We have shown that the formation of localized labyrinthine patterns is not specific to particular plants or soils. We have found localised labyrinths in ecosystems on flat landscapes and hills. Three relevant models which undergo localised vegetation labyrinthine patterns have been considered; (i) vegetation interaction-redistribution model of vegetation dynamics, which can generate patterns even under strictly homogeneous and isotropic environmental conditions. It is grounded on a spatially explicit formulation of the balance between facilitation and competition. Ecosystems experience transitions towards landscape fragmentation of landscapes (ii) the nonvariational Swift–Hohenberg model that can be derived from the model (i) in the long-wavelength pattern forming regime, and (iii) reaction–diffusion model that incorporates explicitly water transport. We have shown that all these models despite their mathematical structure support the phenomenon of the localised labyrinth. We have established their bifurcation diagram and identified a parameter region, where we have observed a coexistence between a homogeneous cover and an extended labyrinthine structure which are both linearly stable. Within it, there exist a pinning zone of parameters where localised labyrinthine vegetation patterns have been generated as a stable pattern. Note however that localised labyrinth is determined by the initial condition, while their maximum peak biomass remains constant for a fixed value of the system parameters. This phenomenon results from front pinning between qualitatively different coexisting vegetation states. Outside of the pinning region, we have shown that the localised labyrinth either shrink and leads to the formation of regular distribution of circular spots or expand leading to the formation of an extended labyrinth. Finally, we have investigated the formation of localised labyrinth on a hill by considering an inhomogenous aridity parameter. This forcing acts as a trapping potential for the labyrinthine pattern. Owing to its general character, robust localised labyrinthine structures observed and predicted in our analysis should be observed in other systems of various fields of natural science such as fluid mechanics, optics, and medicine.

We have documented for the first time the phenomenon of localised vegetation labyrinth by remote observations, using the Google Earth computer program, and numerical simulations of three different theoretical models which are based on ecologically realistic assumptions. These models provide a clear explanation of how nonlinear plant-plant interactions and the effects of plants on soil water can be crucial in determining the spatial distribution of plant communities. It is far from the scope of this contribution to provide parameters assessment and comparison between the theoretical predictions and the field observations. Work in this direction is in progress.

Extended and localised vegetation labyrinthine patterns opens a whole new area of research in self-organisation in vegetation pattern formation, where field observations will be fundamental to establish a connection with the concepts developed in this work.

## Methods

### Google Earth data

The satellite images (cf. Fig. 1) are retrieved from the open-access program Google Earth (see the link https://earth.google.com/web/ and information there), courtesy of CNES/Airbus, Landsat/Copernicus, and Maxar Technologies (Fig. 1a), and CNES/Airbus (see Fig. 1b–d).

The elevation profiles in Fig. 2 are obtained from Google Earth. This software uses digital elevation data from the Shuttle Radar Topography Mission at a resolution of 30 m^44,45^. The error, at a $90\%$ confidence level, associated to the absolute height data is less than 6 m for the territories considered here (Africa and Australia)^44^.

### Climate data

Localised labyrinthine patterns are observed in Central Cameroon (Fig. 1a), Western Australia (Fig. 1b), and Southwest Niger (Fig. 1c, d). The climate types of these regions are humid, arid, and semi-arid, respectively. The climatic classification is based on the aridity index (see Table 1), which is the ratio of mean annual precipitation and potential evapotranspiration^46^. Note that the aridity index is small (big) when the aridity parameter ($\eta$ or $\mu$), defined in the interaction-redistribution approach subsection, is big (small).

### Numerical simulations data

Numerical simulations of models under consideration were solved in square grids with Runge-Kuttta 4 time integrator. The spatial derivatives were approximated using finite difference scheme with a three point stencil using periodic boundary conditions. In the integrodifferential simulation, the convolution integrals were solved in Fourier space through DFT algorithms. The detail of the parameters used in the numerical simulations are listed in the Tables below.

**Table 2:** List of parameter values of the simulations of the non-variational Swift–Hohenberg equation, shown in Fig. 3a ($200 \times 200$ grid, $\eta =1.01$), Fig. 4a ($120 \times 120$ grid, [i, ii, iii] with space step $\Delta x=0.5$), and Fig. 6 ($120 \times 120$ grid).

Cooperativity ($\kappa$)	$\nu$	$\gamma$	$\alpha$	Time step ($\Delta t$)	Space step ($\Delta x$)
0.6	0.011	0.5	0.125	0.05	0.8

**Table 3:** List of parameter values of the simulations of the integrodifferential model, shown in Fig. 3b ($512\times 512$ grid, $\mu =1.301$), Fig. 4b ($256\times 256$ grid), and Fig. 5 ($256\times 256$ grid).

Competition length ($L_{c}$)	Diffusion (*D*)	Facilitation strength ($\xi _{f}$)	Competition strength ($\xi _{c}$)	Time step ($\Delta t$)	Space step ($\Delta x$)
2.5	1	3	1	0.1	0.8

**Table 4:** List of parameter values of the simulation of the reaction–diffusion model, shown in Fig. 3c ($512\times 512$ grid).

$\gamma$	$\omega$	$\theta$	*p*	$\rho$	$\delta$	$\beta$	Time step ($\Delta t$)	Space step ($\Delta x$)
1.45	1.5	0.2	0.7	1.5	100	2.7	0.001	0.6

### Generation of numerical localised labyrinthine patterns

**Figure 7:**
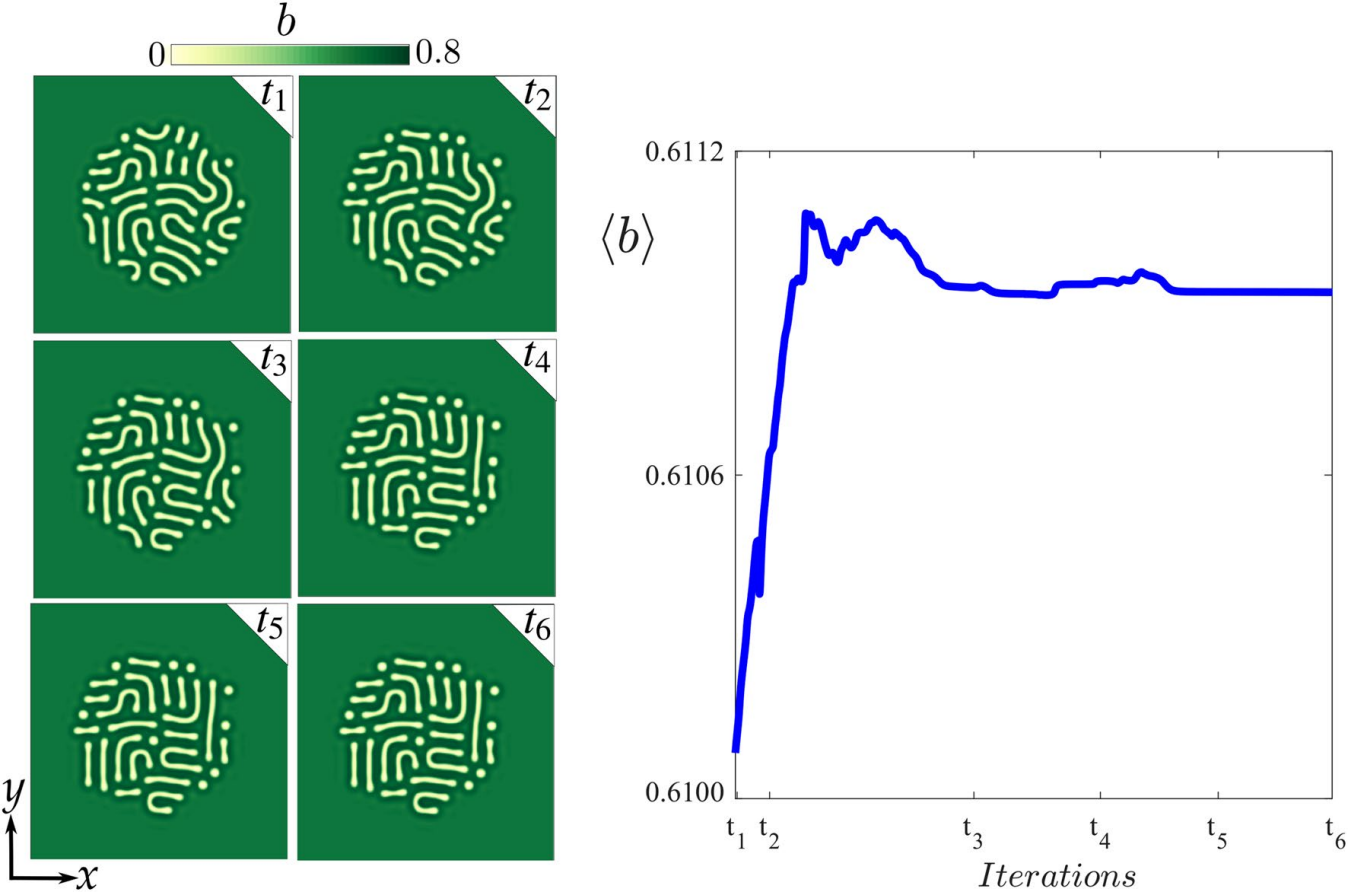
Initialization and stabilization of localised labyrinthine pattern in the integrodifferential model Eq. (2). The aridity parameter is $\mu =1.3$. The other parameters are summarized in Table 3 on "Methods" section. The sequence $t_{1}=1$ to $t_{6}=4\cdot 10^{5}$ accounts for the evolution towards equilibrium of the localised labyrinthine pattern, starting from a circular patch of a labyrinth state embedded in a vegetated background ($t_{1}$). The curve in the right shows the evolution of the average biomass density $\langle b\rangle$, that is the double integral of the two dimensional biomass field *b* divided by the area of the simulation box (see the Integrodifferential model subsection). The stable labyrinthine pattern is reached in $t_5\approx 10^{5}$ iterations of the RK4 time integrator, when there is no change in $\langle b\rangle$. From numerical simulations, the figure was created using Inkscape 1.0 (https://inkscape.org/release/inkscape-1.0/).

The localised labyrinthine patterns are initialised in a region of parameters where the uniform vegetation cover and the labyrinthine pattern coexist, in particular, in a pinning zone (see Fig. 4). The initial condition consists of a circular patch of labyrinthine pattern in the centre of the simulation box, embedded in a homogenous background (see Fig. 7). After a transient accommodation of the biomass field, the stable localised labyrinth emerges. The dynamics towards equilibrium in the integrodifferential model Eq. (2) is resumed in Fig. 7 and the Supplementary Video S1.

### Computation of the bifurcation diagrams

The bifurcation diagrams in Fig. 4 were determined with analytical and direct numerical integration techniques of the governing equations. The blue and black curves account for the vegetated state and the bare one, respectively. The curves are solid when the corresponding state is stable, and broken if unstable. The critical points in which the different states change their stability are determined by linear analysis, detailed in the interaction-redistribution approach subsection.

The red curve is the stable branch of labyrinthine patterns, and it is determined by direct numerical integration of the governing equations (using the algorithm explained above). Starting from a vegetated state with a small amplitude noise perturbation, in the region where the uniform vegetation state is unstable, a stable labyrinthine pattern can emerge (see (ii) in Fig. 4). The stability range of the labyrinth state, that is (i) and (iii) in Fig. 4, are found by decreasing/increasing the aridity parameter starting from the labyrinthine pattern (see the black arrows in Fig. 4).

The blue triangles account for the stable branch of the localised labyrinthine pattern. The initial condition is a stable localised labyrinth state (cf. state (iv) in Fig. 4). The aridity is decreased until the localised labyrinthine pattern becomes a localised hexagonal pattern, which determines the left boundary of the pinning region ($\eta _{p}^{-}$ or $\mu _{p}^{-}$). On the other hand, the right boundary of the pinning region ($\eta _{p}^{+}$ or $\mu _{p}^{+}$) is determined by increasing the aridity until the localised labyrinthine pattern invades all the system (see Fig. 6).

## Supplementary Information

Supplementary Information 1.
